# Evolutionary Rate Heterogeneity and Functional Divergence of Orthologous Genes in *Pyrus*

**DOI:** 10.3390/biom9090490

**Published:** 2019-09-16

**Authors:** Yunpeng Cao, Lan Jiang, Lihu Wang, Yongping Cai

**Affiliations:** 1 Key Laboratory of Cultivation and Protection for Non-Wood Forest Trees, Ministry of Education, Central South University of Forestry and Technology, Changsha 410004, China; xfcypeng@126.com; 2 School of Life Sciences, Anhui Agricultural University, Hefei 230036, China; 3 College of Landscape and Ecological Engineering, Hebei University of Engineering, Handan 056038, China

**Keywords:** *Pyrus*, positive selection, selective modes, functional divergence, expression pattern

## Abstract

Negatively selected genes (NSGs) and positively selected genes (PSGs) are the two types of most nuclear protein-coding genes in organisms. However, the evolutionary rates and characteristics of different types of genes have been rarely understood. In the present study, we investigate the rates of synonymous substitution (Ks) and the rates of non-synonymous substitution (Ka) by comparing the orthologous genes of two sequenced *Pyrus* species, *Pyrus bretschneideri* and *Pyrus communis.* Subsequently, we compared the evolutionary rates, gene structures, and expression profiles during different fruit development between PSGs and NSGs. Compared with the NSGs, the PSGs have fewer exons, shorter gene length, lower synonymous substitution rates and have higher evolutionary rates. Remarkably, gene expression patterns between two *Pyrus* species fruit indicated functional divergence for most of the orthologous genes derived from a common ancestor, and subfunctionalization for some of them. Overall, the present study shows that PSGs differs from NSGs not only under environmental selective pressure (Ka/Ks), but also in their structural, functional, and evolutionary properties. Additionally, our resulting data provides important insights for the evolution and highlights the diversification of orthologous genes in two *Pyrus* species.

## 1. Introduction

It is well known that neutral genes, negatively selected genes (NSGs), and positively selected genes (PSGs) are the three types of all nuclear protein-coding genes in organisms [1]. By comparing the rates of synonymous substitution (Ks) and the rates of non-synonymous (Ka), researchers can identify the differentiating gene type. In general, Ka/Ks > 1 indicates positive selection, and Ka/Ks < 1 is potential evidence for negative selection. Certainly, Ka/Ks = 1 may provide evidence of neutral evolution during gene sequence divergence [2]. However, the evolutionary forces of non-synonymous and synonymous substitution rates are still excluded. Fortunately, previous studies have elucidated their relationship with the physical location of gene, gene type, mutation rates, and guanine–cytosine (GC) content [3,4,5,6,7,8]. These studies also provided a basis for our comparisons of the functional characteristics of the orthologous genes between *Pyrus bretschneideri* Rehd. Dangshansuli and *Pyrus communis* L. Bartlett.

To further understand the gene frequencies under different selection patterns, previous studies were performed on the rate of divergence between *Drosophila* and its close relatives [9,10]. The evolution of non-reproductive-related proteins was relatively slower than reproductive proteins [9,10]. More than 10% of male reproductive proteins have higher Ka/Ks values (i.e., Ka/Ks > 1) [11], the best explanation of which is positive selection [12,13]. In primates, researchers also found similar results [14]. Other strong candidates for positive selection (i.e., fast-evolving genes) were found, such as immune system genes, major histocompatibility complex genes, and mammalian olfactory receptors [15,16,17,18]. In the genome, the overall PSGs accounted for only 0.5% to 5.3% of the whole gene [19]. This is reasonable because mutations in non-synonymous sites are largely considered harmful, so most of them will be rapidly lost during evolution, which leads to lower Ka and lower Ka/Ks ratio. This largely explains why most genes are always evolving under purification/negative selection [3,20,21,22,23]. Compared to animal studies, the genome-wide analysis of gene variation rates is still quite limited in plants, mainly because most higher plants have experienced one or more genome-wide duplication events [24], which leads to a difficult identification of the real orthologous gene [2]. So far, some studies have been conducted on evolutionary rates using orthologous genes of *A. thaliana* and *A. lyrata* [25,26], of *Brassica rapa* and *Brassica oleracea* [3]*,* and of *G. max* and *G. soja* [4]. These results were helpful to understand the underlying mechanisms in which evolutionary rates and genes were interrelated. However, the functional divergence in orthologous genes of *P. bretschneideri* and *P. communis* has not been studied. Therefore, the evolutionary fate of these orthologous genes that evolved from the same ancestor is largely unknown.

As major members of the Rosaceae family, *P. bretschneideri* and *P. communis* are not only the third kind of fruit of economic plants after apple and grape, but also have important ornamental value. Recently, whole genome sequencing work for these two kinds of pears has been completed [27,28], providing useful data material for the comparative genomics research between similar species. *P. bretschneideri* and *P. communis* diverged between 6.6 and 3.3 million years ago (MYA) [29]. The genome size of *P. bretschneideri* [27] and *P. communis* [28] is 527 Mbp with 42,341 genes and 577 Mbp with 43,419 genes, respectively. Comparative genomics studies indicate that the pear genome has undergone two genome-wide duplication events [27]. Moreover, chromosomal evolution studies show that nine ancestral chromosomes are not only the origin of the Maloideae, but also the ancestors of the whole Rosaceae family [27]. In terms of fruit flesh quality, *P. communis* has melted flesh, while *P. bretschneideri* has crisp flesh. The pathways that affect their flesh may be lignin and sorbitol metabolic pathways [27,29,30,31,32]. Therefore, the identification of orthologous genes and the expression analysis of the pear during fruit development stage may help further understanding of the difference in fleshy qualities between *P. communis* and *P. bretschneideri*, and provide a new way to improve the quality of pear fruit.

## 2. Materials and Methods

### 2.1. Data Source and Orthologous Estimation

The genome sequences and gene function annotation files for Chinese pear (*P. bretschneideri*) and European pear (*P. communis*) were obtained from the Pear Genome Project (http://peargenome.njau.edu.cn/), and GDR databases (https://www.rosaceae.org), respectively. The *P. communis* genome was used as a reference, and then the orthologous genes of *P. bretschneideri* and *P. communis* were determined by MCScan (version 1.1) [33] with an E-value of 1 × 10^−5^. MUSCLE (version 3.8.31) was used to execute the protein alignments with default parameters [34]. Kaks_calculator (version 2.0) was used to estimate the Ka, Ks, and Ka/Ks values with NG method [35].

### 2.2. Gene Structure Analysis

Frequency of optimal codons (FOP) is the ratio of optimal codons to synonymous codons. Codon bias index (CBI) is a measure of codon usage bias according to the codon usage of a specific reference set of genes. Codon adaptation index (CAI) is a measure of the relative adaptiveness for each codon with respect to the codon usage of a reference set of highly expressed genes. We calculated statistics, including exon number, exon length, FOP, CBI, and the CAI, as estimated by CodonW (version 1.4.4) (http://www.mybiosoftware.com/codonw-1-4-4-codon-usage-analysis.html) with preferred codons in *P. bretschneideri* using default parameters.

### 2.3. Expression Profile Analysis

To further understand the functional divergence of orthologous gene pairs during pear fruit development, the raw RNA-seq reads from both of *P. bretschneideri* and *P. communis* fruit development were downloaded from the SRA (short-read archive) database of NCBI (PRJNA299117). The pipeline Fastq clean was used to remove low-quality base-calls (minimal mean Phred quality 20) of raw RNA-seq reads and trimmed [36]. The pipeline Tophat2 (version 2.1.0) was used to map clean readings to the *P. bretschneideri* and *P. communis* reference genome, respectively [37]. TopHat2 (version 2.1.0) parameters “read gap length”, “read edit distance” and “allowed mismatches” were set at 4. Cufflinks (version 2.2.1) was used to detect the differentially expressed genes using FPKM (Fragments Per Kilobase per Million) with default parameters [38]. R (version 3.4.1) was used to draw the heatmap of the orthologous gene pair expression [39].

### 2.4. Statistical Tests and Functional Divergence Analysis

SPASS (version 22.0) was used for statistical analysis. Pearson’s correlation coefficient was used to evaluate the similarity between the expression profiles of each orthologous gene pair. We proposed significant values to check the degree of expression diversity: i.e., r > 0.5 for non-divergence, 0.3 < r < 0.5 for ongoing-divergent, and r < 0.3 for divergence [40,41].

## 3. Results

### 3.1. Identification of Orthologous Gene Pairs

It is well known that orthologous gene pairs may have similar functions. To detect the orthologous gene pairs of *P. bretschneideri* and *P. communis*, MCScan software was executed with E-value cut off 10^−5^. Subsequently, we observed the near-linear distribution of homologous regions for all 17 corresponding chromosomes between both pear genome of *P. bretschneideri* and *P. communis* (Figure S1). Ultimately, we identified 6422 orthologous gene pairs (Figure S2) and found that they belong to 630 homologous blocks. Remarkably, we found that the nucleotide sequences of 259 orthologous gene pairs were identical, so these gene pairs were excluded from further analysis.

### 3.2. Distribution of Ka/Ks, Ks, and Ka, and Their Correlations in Pyrus

In the current study, 6163 orthologous gene pairs of both pear genome of *P. bretschneideri* and *P. communis* were used for analysis. We calculated the Ka, Ks, and Ka/Ks of these orthologous gene pairs by using KaKs_calculator software. The Ks values of 99 gene pairs were more significant than 0.3, so these gene pairs were discarded because of the risk of saturation or misalignment. According to the value of Ks and Ka, 6064 orthologous gene pairs were mainly divided into two categories: 5460 negatively selected genes (Ka/Ks < 1); 323 positively selected genes (Ka/Ks > 1); and no neutrally evolved genes (Ka/Ks = 1) (Table S1). The remaining orthologous gene pairs might represent a specific type of gene set in the *Pyrus* genome because either Ks or Ka, or both, were zero. Based on the values of Ka and Ks, we further speculate that there are three forms of selection of these genes: strongly negative selection (Ka = Ks = 0: meaning these genes are strongly constrained); positive selection (Ka ≠ 0; Ks = 0); and negative selection (Ka = 0; Ks ≠ 0).

**Figure 1:**
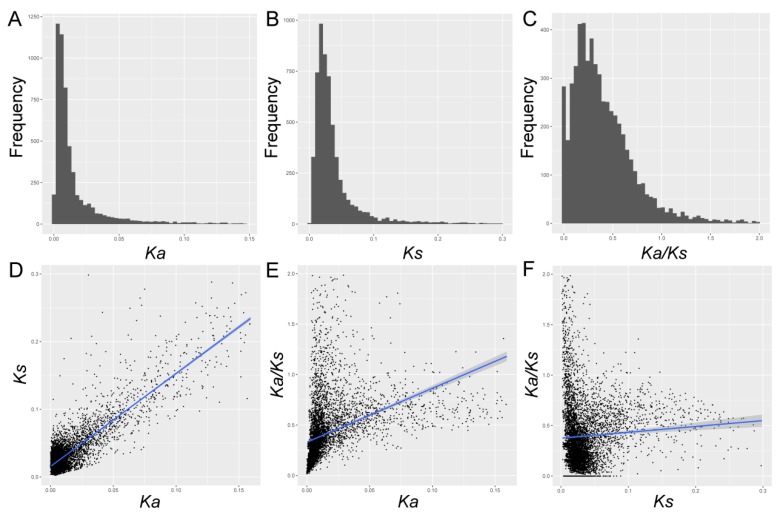
The frequency distributions (**A**–**C**) and correlation analyses (**D**–**F**) of Ka, Ks, and Ka/Ks. (**A**–**C**) The frequency displays of Ka, Ks, and Ka/Ks, respectively. (**D**) The correlation between Ka and Ks. (**E**) The correlation between Ka/Ks and Ka. (**F**) The correlation between Ka/Ks and Ks. The blue line represented the fitted curve, and the shaded part represented the confidence interval.

Ka/Ks, Ks, and Ka were estimated for each gene pair. To sum up, the average value of Ks was 0.019, with a range of 0 to 0.3. The Ka estimates varied from 0 to 0.15, with an average value of 0.011. 90% of Ka/Ks ranged from 0.003 to 0.920, with a mean of 0.271 (Figure 1 and Table S2). In this study, we found that both Ks and Ka values in *Pyrus* were significantly lower (Mann–Whitney U test, *p* < 0.001), while they contained higher Ka/Ks values. These results indicated that these genes might undergo lower selective pressure, evolving at a lower evolutionary rate. Additionally, we also established the relationships of Ka/Ks, Ka, and Ks in *Pyrus*. We found that the Ka increases gradually with the increase of Ks, as shown in Figure 1, with the r = 0.75, *p* < 10^−10^ (Spearman’s rank correlation). This data was basically consistent with that in Brassica (r = 0.14) [3], soybean (r = 0.22) [4], and Arabidopsis (r = 0.21) [26], although there was slight difference in their degree of correlation, suggesting that the mechanisms affecting both Ka and Ks sites might share in different genomes. Additionally, the Ka/Ks ratio was positively correlated with both Ks (r = 0.34, *p* < 10^−10^) and Ka (r = 0.13, *p* < 10^−10^). The correlation between Ka and Ka/Ks was greater than Ks, which indicated that Ka might be a determinant factor for Ka/Ks.

### 3.3. Ka, Ks, and Exon Characteristics Between PSGs and NSGs

**Figure 2:**
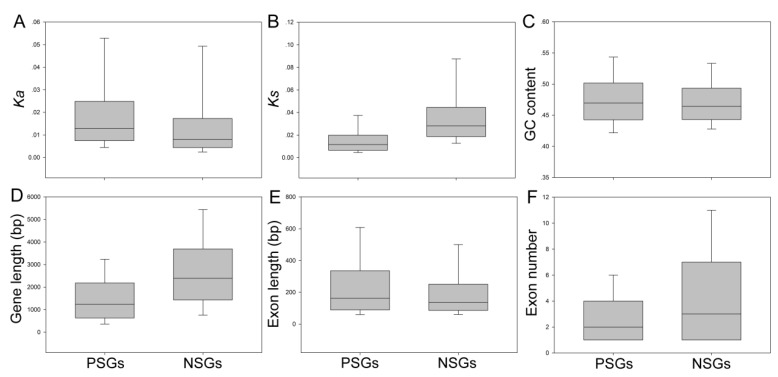
Comparisons of genomic features among different types of negatively selected genes (NSGs) and positively selected genes (PSGs) in *Pyrus*. The top and the bottom of each box were the third (higher) and first (lower) quartiles. The line represented the median value in the box. Out of range, mild outliers are excluded in this figure. (**A**–**F**) The box plot displays of Ka, Ks, GC content, gene length, exon length, and exon number between two selections.

**Figure 3:**
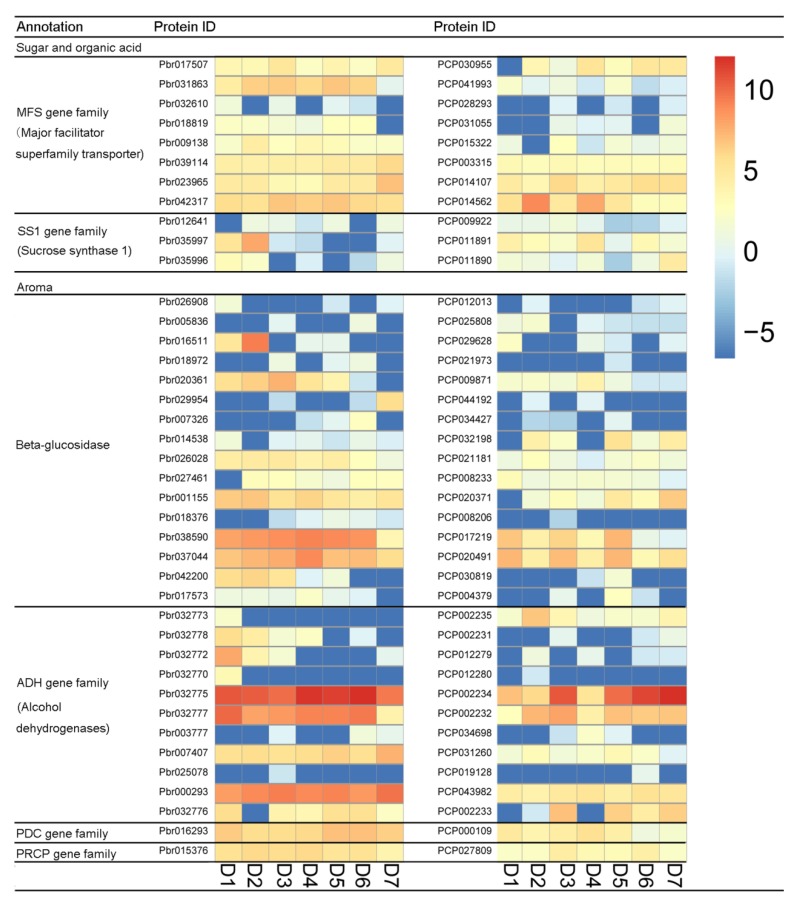
Expression divergence analysis for the related to fruit quality orthologous genes among *Pyrus bretschneideri* and *Pyrus communis.* D1, D2, D3, D4, D5, D6, and D7 indicates 15 days after full blooming (15 DAB), 30 DAB, 55 DAB, 85 DAB, 115 DAB, mature stage, and fruit senescence stage. The higher expression of genes was shown using the red shades and lower expression of genes was shown with the blue shades.

To understand the differences in evolutionary rates between PSGs and NSGs, the Ka and Ks values for each different gene set were estimated separately. The average ratio of Ks for NSGs in *Pyrus* was 0.0276 ± 0.0026, which was two-fold higher than the average ratio of PSGs of 0.0105 ± 0.0076 (Figure 2 and Table S3). On the contrary, we found that the overall Ka for NSGs was 0.0081, which was lower than the overall value of PSGs of 0.0130 ± 0.0083 (Figure 3 and Table S3). These results have also been supported by previous studies [3]. In the present study, to understand the evolutionary rates between NSGs and PSGs, we also investigated the distributions of Ks and Ka. We scanned that the NSGs and PSGs both contain a Ka peak, but the peak of NSGs was much than lower than that of PSGs (Figure S3). On the contrary, most of the Ks values of PSGs were close to 0.01, and most of the Ks values of NSGs are concentrated in the 0.04–0.14 range and some are even higher (Figure S3).

To determine whether gene structure is affected by selective modes and evolutionary rates, we characterized the genetic characteristics for individual *Pyrus* orthologs, such as GC content, exon number, exon length, and gene length (Figure 2). These results revealed that PSGs in *P. bretschneideri* contained fewer exons (average 2 versus 3), higher exon length (average 160.773 bp versus 132.007 bp), and a significantly shorter gene length (average 1255.88 bp versus 2352.33 bp; Mann–Whitney U test, *p* < 0.001) than NSGs (Figure 2 and Table S3). Remarkably, we did not detect any difference in GC content between PSGs and NSGs (Figure 2 and Table S3).

### 3.4. Lower Expression Level for PSGs Than NSGs

The evolutionary rates were usually associated with gene expression, such as the level of gene expression during pear fruit development. To further understand the difference in expression patterns between NSGs and PSGs, the RNA-seq data were used to estimate each gene expression level during pear fruit development [42]. Meanwhile, the previous studies had indicated that the orthologous genes were classified into two groups, including strongly expressed genes (i.e., FPKM ≧ 50) and weakly expressed genes (i.e., FPKM ≦ 3) [43]. These data indicated that the expression level of NSGs was overall much higher than PSGs (Figure S4). Subsequently, we found that 29.23% of NSGs were weakly expressed, and 9.97% of them contained a high expression level. On the contrary, only 4.26% of PSGs were highly expressed, and 55.4% of them were expressed at a very low level (Table S4). In general, the Ka/Ks values of both between PSGs and NSGs were different, so we have explored the correlation between the Ka/Ks values and the expression patterns. First, the Ka/Ks values from *P. bretschneideri* to *P. communis* orthologous and correlated them with expression level were collected. The significantly negatively correlated was found among the Ka/Ks values and expression level. Because the Ka/Ks has a certain relationship with Ka and Ks, so we concluded that the expression patterns were related to them (i.e., Ka, Ks and Ka/Ks). These results are also consistent with previous studies on *Brassica* and *Arachis* [3,44].

### 3.5. Codon Bias Analysis of PSGs and NSGs

Codon bias refers to the different use frequency of synonymous codons in a wide variety of organisms [45,46,47]. To gain insight into whether PSGs and NSGs contain codon bias, we estimated the CBI, the CAI, as well as frequency of optimal codons (FOP), respectively. Compared with NSGs, PSGs have shown consistently higher codon bias for the three codons (i.e., CBI, CAI, and FOP) bias parameters as shown in Table S5. To determine the relationship between codon bias and genetic characteristics, we performed correlation analysis between them. There was a correlation between CBI, CAI, and Ka/Ks. Exon length and/or gene length were negatively correlated with CAI, FOP, and/or CBI. There was a positive correlation between exon number and CAI but negatively correlated with CBI and FOP. GC content was negatively correlated with CAI but was positively correlated with CBI and FOP. There was a positive correlation between expression level and CBI but was negatively correlated with CAI and FOP (Table S6).

### 3.6. Gene Expression Patterns in Pyrus Fruit Revealed Subfunctionalization and Functional Redundancy for the Related to Fruit Quality Genes

Orthologous gene pairs may have similar expression profiles. To detect the degree of expression diversity between orthologous genes in *P. bretschneideri* and *P. communis*, their expression correlations were calculated. We found 25.6% orthologous gene pairs to be non-divergent, and 12.7% of orthologous gene pairs to be ongoing-divergent (Table S7). In combination with these analyses, it was found that significant functional divergence has occurred of orthologous genes between both *P. bretschneideri* and *P, communis*.

Previous studies have shown that sugar, aroma, organic acid, and lignin are important factors affecting the quality of pear fruit [32]. *P. bretschneideri* and *P. communis* fruit quality differs. For example, *P. communis* has melted flesh, and *P. bretschneideri* has crisp flesh. To explore the effect of orthologous genes on pear quality, this study identified the gene families related to pear quality, such as *MFS* gene family, *ADH* gene family, and *PDC* gene family (Table S8). Subsequently, the expression profiles of these gene family members were analyzed in both *P. communis* and *P. bretschneideri* fruit. In the *MFS* gene family, we found that all eight orthologous gene pairs were divergent (Figure 3). Remarkably, *Pbr031863* has shown high expression levels in almost all periods, while its corresponding orthologous gene (*PCP041993*) exhibits low levels of expression. In the *SS1* gene family, we found that all three orthologous gene pairs were divergent, and only one duplicate gene pair (*Pbr037395*/*PCP029644*) was found to be non-divergent. In the *Beta-glucosidase* gene family, three out of 16 orthologous gene pairs were ongoing-divergent, seven out of 17 orthologous gene pairs were non-divergent, but the remaining gene pairs were found to be divergent. In the *ADH* gene family, we found 11 out of 19 orthologous gene pairs to be divergent, with two orthologous gene pairs (*Pbr32775*/*PCP002234* and *Pbr032777*/*PCP002232*) showing higher expression levels. Interestingly, the *Pbr32775* and *Pbr032777* were mainly expressed in the early or middle stage of fruit development, and its corresponding orthologous genes (*PCP002234* and *PCP002232*) are mainly expressed at the later stage of fruit development. Additionally, *Pbr016293*/*PCP000109* and *Pbr015376*/*PCP027809* were also found to be divergent, which belong to the *PDC* gene family and *PRCP* gene family, respectively (Figure 3). The lignin content is also an essential factor affecting the taste of pear fruit [48,49], so we also analyzed the expression diversity of the gene family members involved in lignin biosynthesis, such as *PAL*, *C3H*, *PRX*, *4CL*, *HCT*, *CAD*, *COMT*, *CCoAOMT*, and *CCR* genes. In the present study, we found *Pbr008387*/*PCP019735* (which belong to *PAL* gene family members), *Pbr022402*/*PCP013799* (which belong to *CCR* gene family members), *Pbr024792*/*PCP008177* (which belong to *HCT* gene family members) and *Pbr010872*/*PCP026787* (which belong to *HCT* gene family members) were also found to be divergent (Figure S5). Remarkably, *PCP013799* were more highly expressed than *Pbr022402* in almost all periods, indicating these genes that these genes may play an essential role during pear fruit development.

## 4. Discussion

Surprisingly, there are few studies to describe the evolutionary rate variation and gene expression diversity by comparing orthologous gene pairs, which closely related to plant nuclear genes. Several current studies on this aspect are relatively small samples, but small sample studies are exclusive. For example, Tiffin and Hanada (2009) analyzed 218 orthologous gene pairs between *Brassica rapa* and *Arabidopsis thaliana* [50]; Zhang et al. (2002) studied 242 paralogous gene pairs in *Arabidopsis thaliana* [2]; Wright et al. (2004) identified 83 orthologous between *Arabidopsis thaliana* and *Arabidopsis lyrata* [51]. Recently, several large sample studies have been implemented; for example, Guo et al. (2017) analyzed 23,817 orthologous gene pairs between *Brassica rapa* and *Brassica oleracea* [3]. Although several studies have characterized the functional relationships among multiple species of orthologous gene pairs [52]. Up to now, there have been no studies on the functional features of orthologous genes of *P. bretschneideri* and *P. communis*. Additionally, we also explored the reasons for the differences in the quality of pear fruit from the transcriptome level for the first time.

In the present study, the *Pyrus* species were used as a fruit model system, and 5978 orthologous gene pairs were identified between *P. bretschneideri* and *P. communis*. The Ka/Ks analysis showed that these genes could be divided into two types, namely NSG (Ka/Ks < 1) and PSG (Ka/Ks > 1). Subsequently, we observed several interesting phenomena, including: (a) PSGs in *Pyurs* demonstrated four-fold lower Ks values and two-fold higher Ka; (b) PSGs contained two-fold fewer exons and two-fold shorter gene/exon lengths than NSGs; (c) PSGs genes were very weakly expressed during pear fruit development than NSGs; (d) Gene expression patterns indicated that orthologous genes have different functions, which might be responsible for differences in fruit quality between *P. bretschneideri* and *P. communis*. Our results show that NSGs and PSGs were not only in the selection pressure but also in gene characteristics, evolutionary rates and expression patterns were also different and were consistent with the results of previously published papers [3]. These data indicate that such selective patterns might be shared in some plants, such as pear, *Brassica rapa*, and *Arabidopsis thaliana*.

Previous studies have shown that the Ks of NSGs is much higher than PSGs [3]. In the present study, this phenomenon was also found, which might be due to PSGs with strong codon bias being weakly expressed. These properties might reduce the synonymous mutation rates of PSGs, which ultimately lead to smaller Ks values. This might also be the stronger codon usage that could improve the efficiency of translation since the use of codons that match most tRNA can reduce the time between finding and binding the correct tRNA [53]. In addition, we found that PSGs contained much shorter exon length and gene length than NSGs. These results have also been verified in *Brassica rapa*, *Arabidopsis thaliana*, *Caenorhabditis elegans*, and *Drosophila melanogaster* [3,40,53]. Remarkably, there is a strong negative correlation between protein length and codon usage, further supporting the previous view that the selective model may be a key factor for gene properties. Some of the previous studies have explained the relationship between gene expression and gene structure. For example, these genes, which contained shorter, fewer introns/exons and shorter coding region, were highly expressed in humans [3,54,55,56,57]. According to these studies, three factors (i.e., genomic design, mutation bias and transcriptional efficiency) could explain the compact gene structure. In monocot species of *O. sative* and dicot species of *A. thaliana*, highly expressed genes were reported to contain longer gene transcripts [58]. The resulting contrasts between plants and animals could be explained by the outcome of selective forces and different turns after their splits [58]. Since the correlation of gene structure and gene expression was different among different genomes, the elective modes might serve as an alternative indicator for gene compactness, as proposed in the present study. As shown in Table S1, PSGs in *P. bretschneideri* contained shorter gene length and lower intron/exon numbers than NSGs. The pattern analyses of positive selection were carried out in mammalian genomes [59]. Previous studies have shown that expression patterns of PSGs in mammals and plant genomes were very similar, such as expressed at lower levels [3,59]. In fact, the relationship between gene characteristics and selective modes still requires more genomic data to verify. Fruit development and ripening involves a series of physiological and biochemical changes, which was a highly coordinated and irreversible biological process [32]. Previous studies reported that the composition and content of soluble sugars were significant factors affecting pear fruit quality [32,33,34]. It is well known that there are differences in fruit quality between *P. bretschneideri* and *P. communis*. To understand these differences, the related gene families of pear fruit quality were identified, such as *MFS* gene family, *ADH* gene family, and *PDC* gene family. Previous studies suggested that orthologous gene pairs might have similar expression patterns or functions [30,40]. In our research, the degree of expression diversity of orthologous gene pairs was estimated between *P. bretschneideri* and *P. communis*. Subsequently, we found that most orthologous gene pairs were diverged. Among them, some genes were expressed more highly in *P. bretschneideri*, compared to their orthologous genes in *P. communis*, such as *Pbr000274* and *Pbr024748* (which belong to *MFS* gene family members), *Pbr007408* and *Pbr000293* (which belong to *ADH* gene family members) etc. At the same time, similar genes are also found in *P. communis*, such as *PCP020491*, *PCP041912* (which belong to *MFS* gene family members). These expression patterns revealed functional redundancy for some orthologous genes derived from a common ancestor and subfunctionalization for some of them. The present study also might help us to further explore the differences between the fruit quality of *P. bretschneideri* and *P. communis*. In conclusion, our finding provides a strong foundation for future research on gene function and breeding, which will help improve fruit quality.

## Supplementary Materials

The following are available online at https://www.mdpi.com/2218-273X/9/9/490/s1, Figure S1: Inter-genome synteny for the *Pyrus bretschneideri* and *Pyrus communis*. Synteny between *Pyrus bretschneideri* and *Pyrus communis* shows 6422 gene pairs, Figure S2: Synteny relationships of orthologous gene pairs between *Pyrus bretschneideri* and *Pyrus communis*. *Pyrus bretschneideri* and *Pyrus communis* chromosomes were represented by yellow and green, respectively. The grey lines indicated orthologous relationships between *Pyrus bretschneideri* and *Pyrus communis*. Figure S3: Density distributions Ka (a), Ks (b), GC content (c) and gene length (d). Positively selected genes (PSGs) and negatively selected genes (NSGs) were represented by red and green lines, respectively. Figure S4: Frequency distributions of expression levels in *Pyrus communis* during fruit development. Figure S5: Expression divergence analysis of orthologous lignin-related genes among *Pyrus bretschneideri* and *Pyrus communis.* D1, D2, D3, D4, D5, D6 and D7 indicates 15 days after full blooming (15 DAB), 30 DAB, 55 DAB, 85 DAB, 115 DAB, mature stage, and fruit senescence stage. Table S1: Features of Ka/Ks in 6064 orthologs between *Pyrus bretschneideri* and *Pyrus communis.* Table S2: Evolutionary rates for 6064 orthologs between *Pyrus bretschneideri* and *Pyrus communis.* Table S3: Comparisons between positively selected genes (PSGs) and negatively selected genes (NSGs) in *Pyrus bretschneideri.* Table S4: Comparisons of expression patterns between positively selected genes (PSGs) and negatively selected genes (NSGs) in *Pyrus bretschneideri*. Table S5: Codon bias comparisons between PSGs and NSGs in *Pyrus bretschneideri.* Table S6: Correlation analysis between codon bias and gene properties in *Pyrus bretschneideri*. Table S7: Functional divergence analysis of orthologous genes among *Pyrus bretschneideri* and *Pyrus communis.* Table S8: Functional annotation of PSGs and NSGs in *Pyrus bretschneideri*.
