# Supplementary material for: Evolutionary Rate Heterogeneity and Functional Divergence of Orthologous Genes in Pyrus
Source: Biomolecules. 2019 Sep 16;9(9):490. doi: 10.3390/biom9090490 (PMC6770726; doi:10.3390/biom9090490)
Supplement: Supplementary file 1 [file biomolecules-09-00490-s001.zip › biomolecules-522349-supplementary/Figure S1-6.pdf]

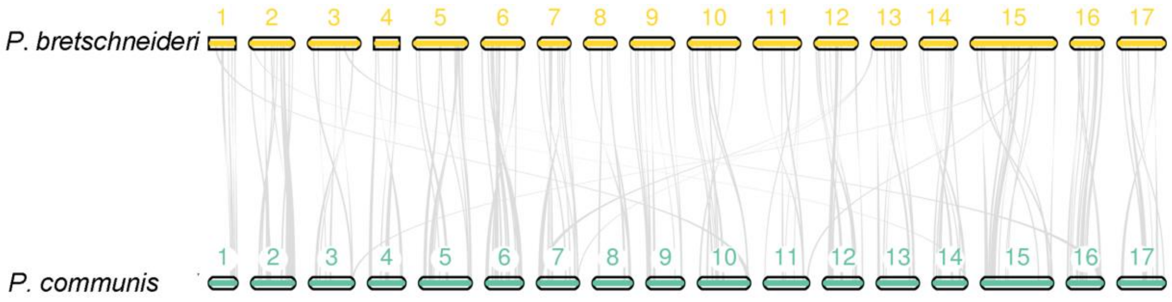

Figure S1. Inter-genome synteny for the *Pyrus bretschneideri* and *Pyrus communis*. Synteny between *Pyrus bretschneideri* and *Pyrus communis* shows 6422 gene pairs.

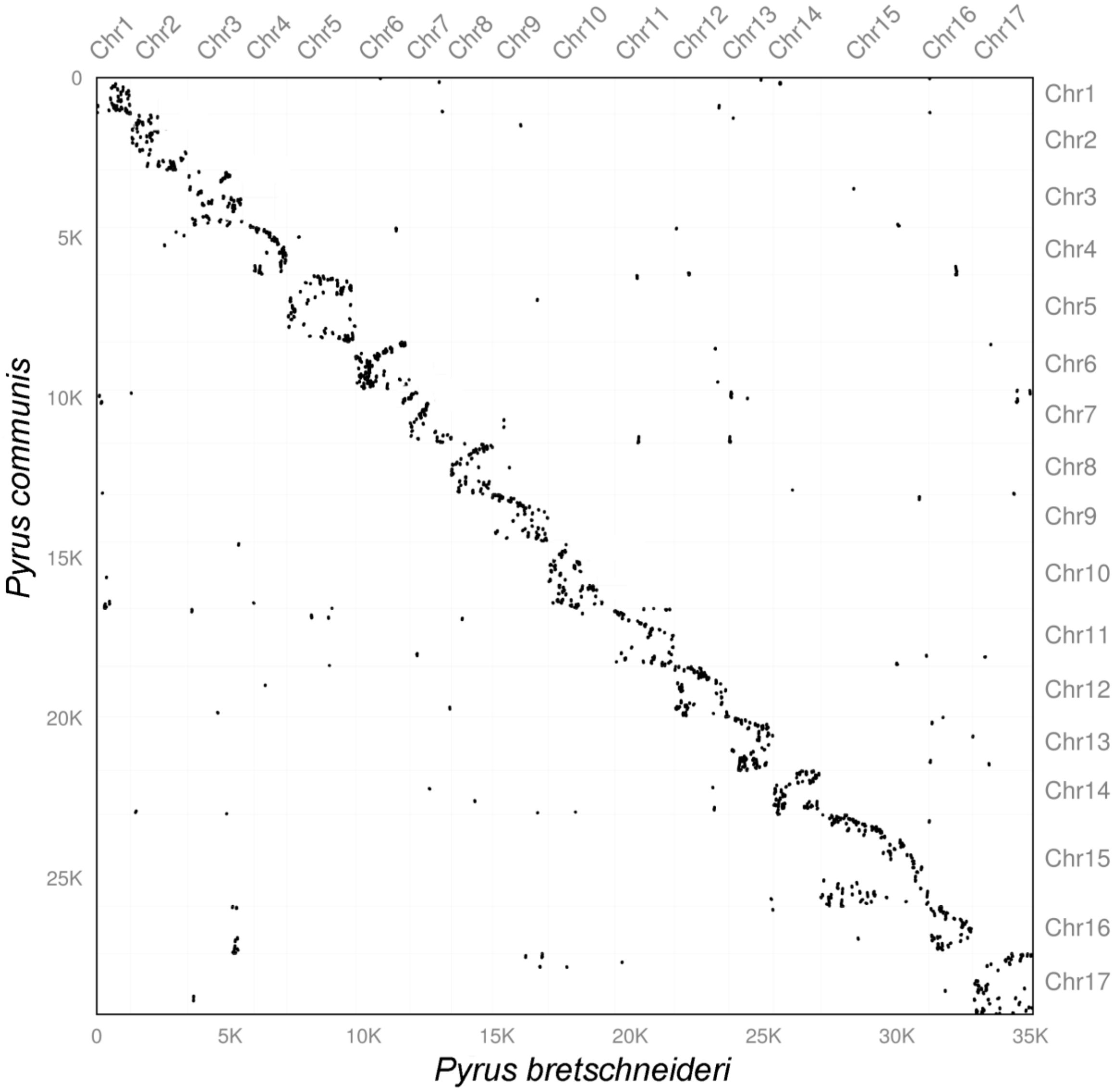

Figure S2. Synteny relationships of orthologous gene pairs between *Pyrus bretschneideri* and *Pyrus communis*. *Pyrus bretschneideri* and *Pyrus communis* chromosomes were represented by yellow and green, respectively. The grey lines indicated orthologous relationships between *Pyrus bretschneideri* and *Pyrus communis*.

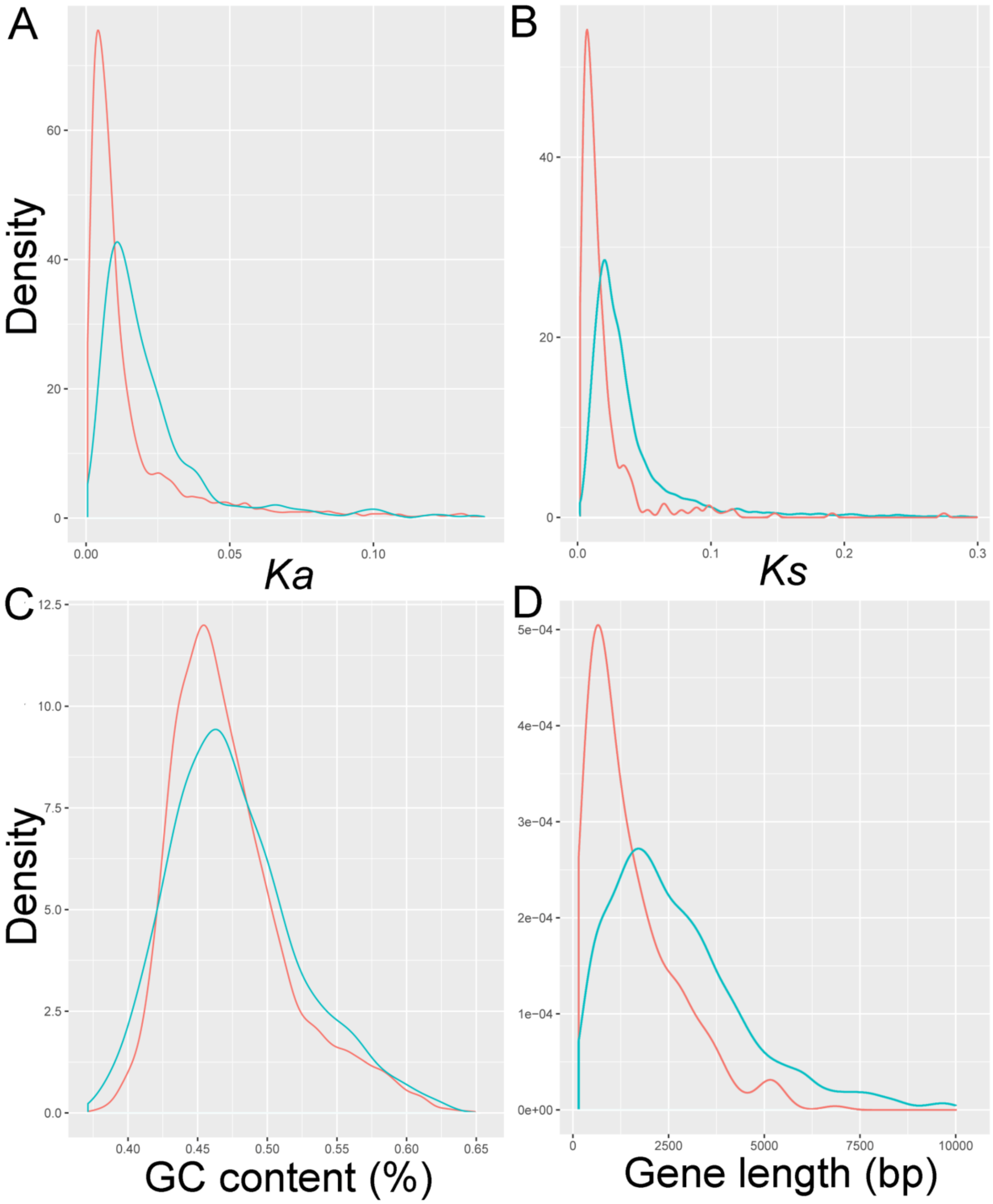

Figure S3. Density distributions Ka (a), Ks (b), GC content (c) and gene length (d). Positively selected genes (PSGs) and negatively selected genes (NSGs) were represented by red and green lines, respectively.

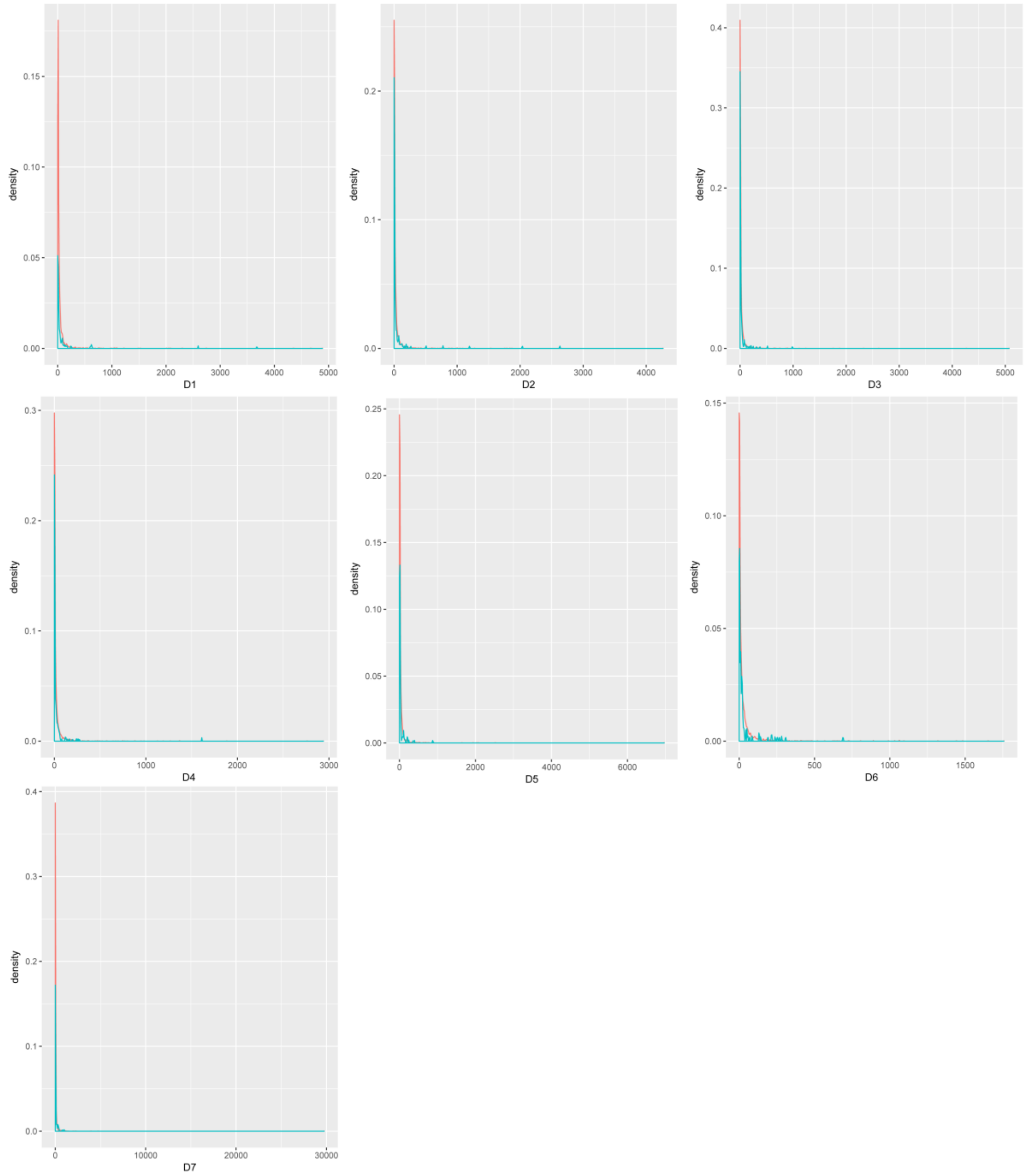

Figure S4. Frequency distributions of expression levels in *Pyrus communis* during fruit development.

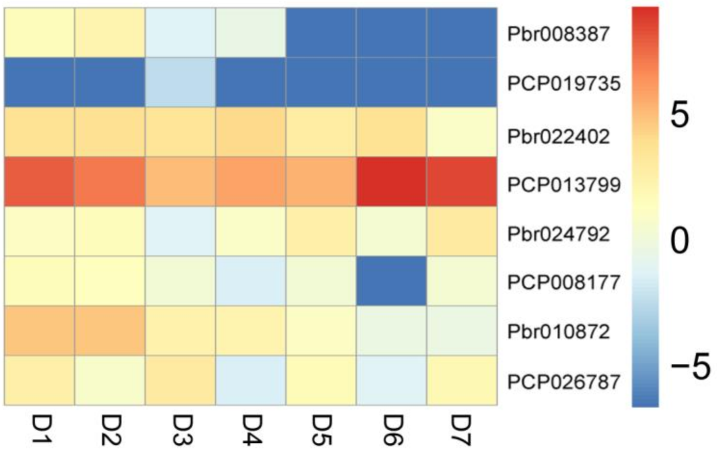

Figure S5. Expression divergence analysis of orthologous lignin-related genes among *Pyrus bretschneideri* and *Pyrus communis.* D1, D2, D3, D4, D5, D6 and D7 indicates 15 days after full blooming (15 DAB), 30 DAB, 55 DAB, 85 DAB, 115 DAB, mature stage, and fruit senescence stage.
